# Eco-Friendly ZnO Nanomaterial Coatings for Photocatalytic Degradation of Emerging Organic Pollutants in Water Systems: Characterization and Performance

**DOI:** 10.3390/nano16010023

**Published:** 2025-12-24

**Authors:** Dušica Jovanović, Szabolcs Bognár, Nina Finčur, Vesna Despotović, Predrag Putnik, Branimir Bajac, Sandra Jakšić, Bojan Miljević, Daniela Šojić Merkulov

**Affiliations:** 1 Department of Chemistry, Biochemistry and Environmental Protection, University of Novi Sad Faculty of Sciences, Trg Dositeja Obradovića 3, 21000 Novi Sad, Serbia; dusica.jovanovic@dh.uns.ac.rs (D.J.); sabolc.bognar@dh.uns.ac.rs (S.B.); nina.fincur@dh.uns.ac.rs (N.F.); vesna.despotovic@dh.uns.ac.rs (V.D.); 2 Department of Food Technology, University North, Trg Dr. Žarka Dolinara 1, 48000 Koprivnica, Croatia; 3 BioSense Institute, University of Novi Sad, Dr Zorana Đinđića 1, 21000 Novi Sad, Serbia; branimir.bajac@biosense.rs; 4 Scientific Veterinary Institute “Novi Sad”, Rumenački put 20, 21000 Novi Sad, Serbia; sandra@niv.ns.ac.rs; 5 Faculty of Technology, University of Novi Sad, Bulevar cara Lazara 1, 21000 Novi Sad, Serbia; miljevic@uns.ac.rs

**Keywords:** ZnO, coatings, green synthesis, pesticide, pharmaceutical, mycotoxin, wastewater treatment, photocatalysis

## Abstract

The present study targets key limitation ‘separation after the process’ that is responsible for the loss of the photocatalyst in water treatment during heterogeneous photocatalysis. Therefore, eco-friendly nanostructured ZnO coatings were engineered by the doctor blade technique through the immobilization of green ZnO nanomaterials onto alumina substrate. ZnO/BPE 30 and ZnO/BPE 60 coatings were obtained from banana peel extract-based ZnO powder (ZnO/BPE). Likewise, ZnO/GTE 30 and ZnO/GTE 60 were prepared using green tea extract-based ZnO powder (ZnO/GTE). XRD characterization verified hexagonal wurtzite ZnO phase, while HRSEM analysis revealed that the flat surface of ZnO/BPE had rod-like nanostructures below 120 nm, and ZnO/GTE had spherical, porous nanoparticle networks with less than 70 nm. According to UV–vis spectrometry, all four coatings have bandgaps of ~5 eV. The highest efficiency for the solar-driven photocatalytic degradation of emerging organic pollutants was for ciprofloxacin (among pesticides clomazone and tembotrione; pharmaceuticals ciprofloxacin and 17α-ethinylestradiol; and mycotoxin zearalenone) in ultrapure water with the presence of all studied ZnO-based coatings, after 60 min of simulated solar irradiation. Its highest removal (89.1%) was achieved with ZnO/GTE 30, also having good reusability across three consecutive cycles in river water, thus supporting the application of eco-friendly, immobilized ZnO nanomaterials for wastewater treatment and environmental remediation.

## 1. Introduction

Driven by human activities such as power generation, waste disposal, and agriculture, environmental pollution has been escalating since the dawn of industrialization. The release of harmful substances has degraded air quality, contaminated vital water resources, and inflicted lasting damage on the planet’s surface. Beyond harming ecosystems, the pollution has also fueled a global public health crisis, contributing to millions of deaths annually [1]. Among the aforementioned pollution types, water pollution is recognized as the greatest emergency, affecting human health and ecosystems. Due to anthropogenic activities, water bodies are polluted with numerous inorganic and organic pollutants. Among the organics, a few groups are highlighted as being more dangerous, such as pesticides, pharmaceuticals, and mycotoxins due to their persistence, toxicity, and possible accumulation within aquatic organisms [2,3]. Therefore, in this research, the potential removal of five selected organics was examined: two pesticides (clomazone—CLO and tembotrione—TEM), two pharmaceuticals (ciprofloxacin—CIP and 17α-ethinylestradiol—EE2), and one mycotoxin (zearalenone—ZEA).

CLO is a selective herbicide widely used for weed control in crops such as rice, soybeans, and cotton. It has been detected in soil, surface water, and groundwater due to agricultural runoff, posing risks to non-target aquatic organisms and soil microbial communities [4,5,6]. On the other hand, TEM belongs to the triketone pesticides. TEM is most commonly applied on corn fields against broadleaf weeds [7,8]. TEM and its degradation intermediates can have harmful effects on non-target organisms, such as reducing non-specific esterase activity in *Tetrahymena pyriformis* and metabolic activity in *Vibrio fischeri* [9]. CIP is a broad-spectrum fluoroquinolone antibiotic [10], mainly used for the treatment of respiratory and urinary tract infections, as well as skin infections [11]. Due to its widespread and careless use, many bacteria, including *Escherichia coli*, *Salmonella enterica*, *Staphylococcus aureus*, and *Klebsiella pneumoniae* have developed resistance to CIP [12]. Its significant impact on the water environment was well observed after the coronavirus disease 2019 pandemic period, when the increase in its prescription, on the account of treating respiratory infections, caused the increase in concentration in rivers [13,14]. EE2 is a synthetic hormone, a derivative of the natural hormone estrogen, which has found widespread use in contraceptive pills. Only a small portion of the ingested EE2 amount undergoes catabolic pathways in the human body, while the remaining amount is excreted naturally and enters the water environment [15]. The presence of EE2 in water bodies, in the first place, affects aquatic organisms [16,17], but the studies also showed its negative influence on terrestrial animals [18,19]. Finally, ZEA, primarily produced by *Fusarium* fungi, contaminates crops such as maize, wheat, oats, rice, and barley. Its health risks stem from its estrogenic and anabolic properties, which disrupt normal reproductive functions in farm animals [20,21].

Due to the decreasing availability of natural water sources, industrial wastewater must be used after undergoing a purification treatment. For this purpose, research is being conducted globally to make the processes more economical and energy-efficient. Commonly used conventional water treatment techniques, which include different physical, chemical, and biological processes, cannot guarantee a full purification. Hence, advanced chemical and biological processes are being studied for the removal of emerging organics [22]. Bearing in mind the importance of eco-friendliness, advanced oxidation processes (AOPs) are widely examined in the removal of organic pollutants, as they rely on the generation of highly reactive oxygen radicals capable of degrading and potentially mineralizing the emerging organics [23]. Since Fujishima and Honda’s groundbreaking discovery of photoelectrochemical water splitting with semiconductors in 1972, photocatalysis has seen remarkable advancements [24]. Among AOPs, heterogeneous photocatalysis stands out for its sustainability, environmental compatibility, and energy efficiency, utilizing only (sun)light and semiconductor-based photocatalysts. This method is particularly effective in treating wastewater containing persistent, highly complex, and poorly biodegradable pollutants, even at higher concentrations [23].

Photocatalysts have a crucial role in heterogenous photocatalysis. One of the most commonly applied photocatalysts is ZnO—an environmentally friendly, biocompatible semiconductor with a wide bandgap energy. Furthermore, it has a greater ability to absorb a broader range of electromagnetic radiation and it also exhibits significantly high quantum efficiency. On the other hand, despite these advantages, wide bandgap (3.2–3.7 eV) limits its performance in the visible region, leading to the rapid recombination of photogenerated electron–hole pairs [25,26]. Additionally, its photocatalytic activity can be diminished at very low and high pH values due to photocorrosion/dissolving [27].

Bearing in mind the sustainability of heterogenous photocatalysis, the aforesaid drawbacks of ZnO have to be improved. Thus, a great attention is paid to the fabrication of novel, bio-inspired ZnO materials appreciating the principles of nanotechnology [28]. Nanotechnology involves the synthesis, characterization, and investigation of materials within the nanometer range (1–100 nm), where the properties and functions of both natural and engineered systems are defined [29]. Various approaches exist for the synthesis of nanomaterials, which can be broadly categorized into physical, chemical, and biological methods. However, for the purpose of leaving the harmful organic chemicals and non-sustainable experimental conditions aside, green and feasible methods, relying on the usage of various plant extracts as an alternative, should be developed [30]. The role of plant extracts is primarily to reduce metal salts, and to cap and stabilize particles of a new nanomaterial [28]. However, when it comes to the application of various, bio-inspired nanomaterials in photocatalysis, there is another problem, which has to be considered and solved. Namely, after the photocatalytic treatment, the used powder-based catalyst has to be removed and regenerated for further application, which represents an additional problem for scaled-up heterogeneous photocatalysis [31]. Thus, currently, the up-to-date studies are focused on immobilizing the photocatalytic nanomaterials on different substrates or supports to achieve better efficiency and enhance the practical implementation of photocatalysis technology, for instance, by coating TiO_2_ or ZnO onto various surfaces using techniques such as dip coating, spin coating, spray pyrolysis, pulsed laser deposition (PLD), and electrophoretic deposition (EPD). Examples include the following: (i) TiO_2_/Ag composites immobilized on fluorine-doped tin oxide (FTO) glass via spin coating with silica binders, (ii) ZnO nanostructures deposited on sapphire substrates using PLD for the enhanced photocatalytic degradation of organic dyes, and (iii) TiO_2_ films fabricated by EPD on stainless steel or glass substrates, which demonstrated improved photocatalytic performance after sintering [32,33].

In light of the aforesaid, the aim of this study was, firstly, to prepare various coatings on alumina substrate, via the doctor blade technique, using ZnO-based nanomaterials that our research group obtained in the green synthesis using extracts of banana peel (ZnO/BPE) [34] and green tea leaves (ZnO/GTE) [23]. The second goal was to characterize the prepared coatings via X-ray powder diffraction (XRD), high resolution scanning electron microscopy (HRSEM), and UV–vis spectroscopy. Moreover, the photocatalytic activity of the coatings was to be tested in the degradation of CLO, TEM, CIP, EE2, and ZEA under various experimental conditions, using simulated solar irradiation (SSI). Finally, the reusability of the most effective coating was set to be determined along with the influence of the river water matrix.

## 2. Materials and Methods

### 2.1. Materials and Reagents

Photodegradation experiments in this study were conducted using the following: (i) herbicides: CLO (CAS No. 81777-89-1; 98.8%; Sigma−Aldrich, St. Louis, MO, USA) and TEM (CAS No. 335104-84-2; ≥97.83%; LGC Labor GmbH, Augsburg, Germany), (ii) pharmaceuticals: CIP (CAS No. 85721-33-1; ≥98%; Sigma−Aldrich, St. Louis, MO, USA) and EE2 (CAS No. 57-63-6; ≥98%; Sigma−Aldrich, St. Louis, MO, USA), and (iii) mycotoxin: ZEA (CAS No. 17924-92-4; >99.9%; Sigma−Aldrich, St. Louis, MO, USA). Table 1 summarizes key information about the investigated organic pollutants. Working solutions of CLO, TEM, CIP, and EE2 (0.05 mmol/dm^3^) were prepared by dissolving the appropriate mass of the substance in ultrapure water (provided by Adrona water purification system (LPP Equipment AG, Uster, Switzerland)), and then kept in the dark. A working solution of ZEA (0.5 µg/cm^3^) was prepared following a previously reported procedure [23].

α-Terpineol (C_10_H_18_O; *M*_r_ = 154.25; CAS No. 98-55-5; 90%; Sigma−Aldrich, St. Louis, MO, USA) and ethyl cellulose (CAS No. 9004-57-3; viscosity 300 cp; Sigma−Aldrich, St. Louis, MO, USA) were used in the process of coating preparation. The components of the mobile phase for liquid chromatographic analysis were acetonitrile (C_2_H_3_N, ACN; *M*_r_ = 41.05; CAS No. 75-05-8; (i) 99.9%, Sigma−Aldrich, St. Louis, MO, USA (for the analysis of pesticides and pharmaceuticals); and (ii) HPLC gradient grade, Merck, Darmstadt, Germany (in the case of mycotoxin analysis)), 0.1% water solution of the phosphoric acid (H_3_PO_4_; *M*_r_ = 97.99; CAS No. 7664-38-2; 85%, *p.a.*, Sigma–Aldrich, St. Louis, MO, USA), and ultrapure water. The sample of surface water was collected from the Danube River (Novi Sad, Serbia) in April 2025. The physicochemical properties of ultrapure and Danube River water are given in our previous work [34].

### 2.2. Preparation of the Coatings

The procedure for film deposition was adopted from Xu et al. [40], in regard to paste composition using α-terpineol and ethyl cellulose, for both ZnO powders. A mixture of precursors was sonicated for 45 min, after mixing at 65 °C for 15 min. Such a mixture was deposited after 24 h using the doctor blade technique. Films were annealed at only 200 °C to avoid possible peeling off during rinsing, which easily occurs in films annealed at higher temperatures. The effect of different annealing times (30 and 60 min) was explored to determine if photocatalytic efficiency can be improved by thermal treatment of the coatings, while simultaneously keeping them stable both mechanically and chemically. Therefore, two coatings prepared using ZnO/BPE are named ZnO/BPE 30 and ZnO/BPE 60, whilst two coatings prepared using ZnO/GTE are ZnO/GTE 30 and ZnO/GTE 60.

### 2.3. Coatings Characterization Techniques

Coatings were characterized by XRD analysis (Rigaku SmartLab, Tokyo, Japan) in 2*θ* range 20–70°. Coatings were recorded in Brag–Brentano optics setup due to their micron thickness. HRSEM micrographs were obtained using a scanning electron microscope (Apreo 2C, Thermo Fisher Scientific, Germering, Germany) with a field emission electron source. SEM images were recorded at magnifications of 5000 and 50,000 times, on 10 kV and 0.1 nA. UV–vis measurements (Thermo Evolution 600 UV–vis spectrophotometer, Thermo Scientific, Germering, Germany) were performed using the diffuse reflectance accessory DRA EV 600 integrating sphere. Measurements of reflectance were conducted in the range 240–840 nm with the step of 1 nm and speed of 10 nm/min. Spectralon^®^ was used as a reference standard. The bandgap energy of prepared films was determined using the Tauc’s plot [41], measuring their reflectance by UV–vis spectroscopy. The method, based on Kubelka–Munk theory [42,43], was described in our previous study [44]. The bandgap energies of ZnO dispersions were determined in the same manner just using the measured absorption instead of the Kubelka–Munk function, as in [45].

### 2.4. Photodegradation Procedure

A commercial batch photoreactor (TOPT-V, Toption, Xi’an, China, described in detail in our previous research [46]) was used to carry out the photocatalytic degradation experiments of selected organics. Aliquots (50.0 cm^3^) of the target pollutant solution (0.05 mmol/dm^3^) were added to the quartz photochemical cells containing different ZnO/BPE and ZnO/GTE coatings. Each experiment was performed under 60 min of SSI. Prior to SSI, the solutions were stirred for 5 min in dark. Degradation samples were taken (after 0, 5, 10, 20, 30, 45, and 60 min of irradiation) and filtered through syringe filters (i) 0.22 µm, PVDF membrane, Millex-GV, Merck KGaA, Darmstadt, Germany (in the case of pesticides and pharmaceuticals solutions), and (ii) 0.22 µm, cellulose acetate, Amtast, Lakeland, FL, USA (in the case of mycotoxin solution)).

### 2.5. Photocatalyst Reutilization

Three equal consecutive runs (each lasting 60 min) were conducted to test the reutilization of ZnO/GTE 30 coating in the removal of CIP (0.05 mmol/dm^3^) from the river water utilizing SSI. For this, the appropriate mass of the target substrate was dissolved in water from the Danube River. After each cycle, the coating was submerged in ultrapure water, exposed to SSI for 60 min, washed, and dried at room temperature. The same coating was then utilized in the photocatalytic degradation of the new amount of CIP solution.

### 2.6. Analytical Procedures

The removal efficiency of herbicides (CLO and TEM) and pharmaceuticals (CIP and EE2) was followed using a liquid chromatograph with diode array and fluorescence detectors (UFLC-DAD/RF, Shimadzu Nexera, Tokyo, Japan) equipped with a nonpolar Eclipse XDB-C18 column (150 mm × 4.6 mm i.d., particle size 5 μm), whereas the removal efficiency of the mycotoxin (ZEA) was monitored using a high-pressure liquid chromatograph with a diode array detector (Dionex UltiMate 3000 Series, Thermo Scientific, Germering, Germany) and a Hypersil Aqua GOLD column (150 mm × 3 mm i.d., particle size 3 μm). Photodegradation samples of the chosen organics were analyzed under the chromatographic conditions previously described [23,47].

## 3. Results

### 3.1. Characterization of the Coatings

To understand the photocatalytic activity of the prepared coatings, the crystal structure and morphology of the deposited films were analyzed with XRD and HRSEM, followed by bandgap determination by UV–vis spectrometry.

**Figure 1:**
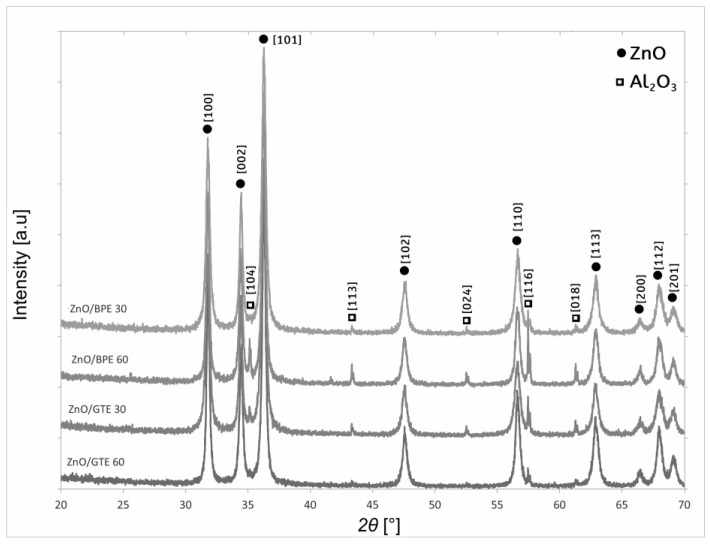
XRD patterns of green-synthesized ZnO/BPE and ZnO/GTE coatings.

The diffraction patterns, shown in Figure 1, generally resemble and confirm a ZnO structure, reflecting crystal plains at 2*θ* positions, characteristic of the hexagonal wurtzite crystal structure of ZnO, with maxima positioned at 2*θ* angles 31.77°, 34.45°, 36.29°, 47.61°, 56.61°, 66.53ׄ°, 68.12°, and 69.25° for crystal planes 110, 002, 101, 102, 110, 113, 200, 112, and 201, respectively [48]. Additional sharp peaks may be observed, with overall lower intensity, assigned to the α-alumina substrate, positioned at 2*θ* angles 35.12°, 43.37°, 52.70°, 57.48°, and 61.43° for crystal planes 104, 113, 024, 116, 018, and 220, respectively, on which ZnO/BPE and ZnO/GTE powders were deposited. Their intensity is much smaller due to the nature of XRD analysis, which analyzes only a few microns of material in depth. This indicates successful preparation in all four cases, in regard to the pure phase composition of ZnO.

**Figure 2:**
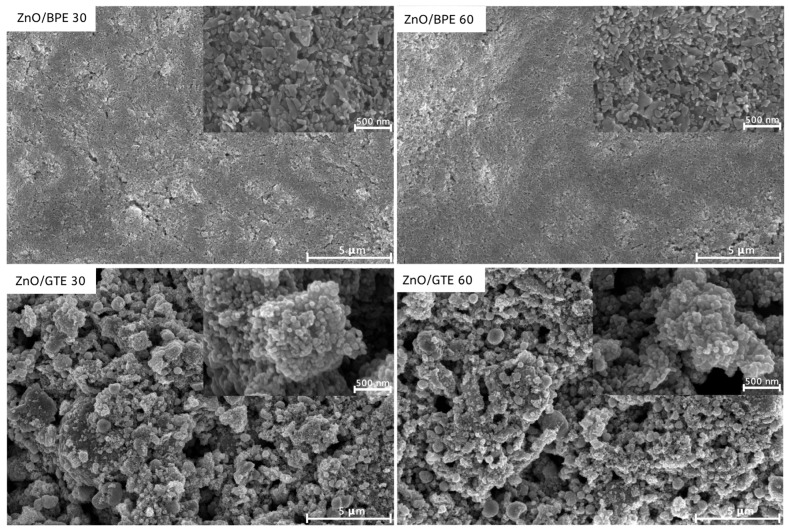
HRSEM micrographs of ZnO/BPE and ZnO/GTE coatings.

Information about the morphology, i.e., surface roughness and the specific surface of the coatings, was obtained after HRSEM analysis (Figure 2). Namely, even with the same deposition procedure applied, ZnO/GTE powder was deposited in the form of a more porous/open structure film, not completely consolidated in a flat homogenous surface on the scale of several microns, unlike ZnO/BPE powder and its two coatings. Rather, the ZnO/GTE film surface was formed from larger clusters of particles, which may be the consequence of the smaller particles found in the ZnO/GTE powder sample.

From the HRSEM micrographs of four coatings at the magnification of 50 k (Figure 2, insets in the upper right corners) it can be seen that the ZnO/BPE samples had particles shaped in plate- or rod-like structures with 50–120 nm in size, whereas ZnO/GTE samples had much more spherical particles (30–70 nm in diameter) with more narrow distribution. Their agglomeration had probably led to such a porous film.

Applying UV–vis spectrometry, absorption spectra were studied and the bandgaps of ZnO/BPE and ZnO/GTE powders were calculated for synthesized powders and deposited films. The procedure and methods of band gap determination via Tauc’s plots are described in Section 2.3.

**Figure 3:**
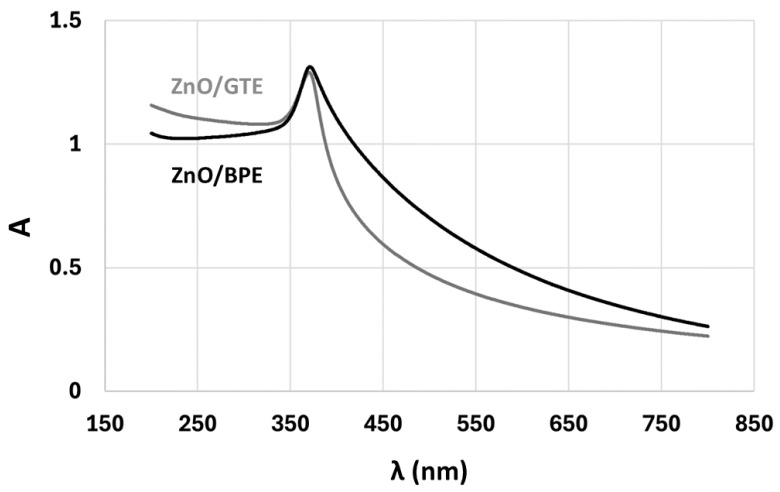
UV–vis absorption spectra of ZnO/BPE and ZnO/GTE powders.

**Figure 4:**
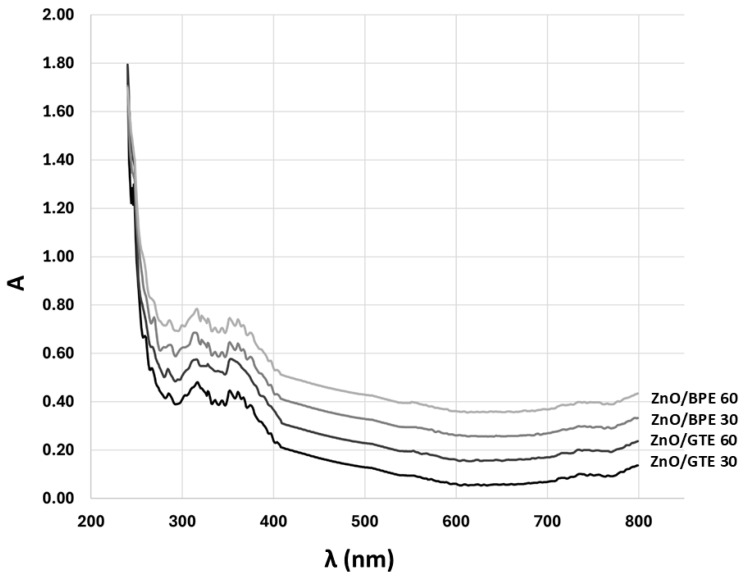
UV–vis absorption spectra of ZnO/BPE and ZnO/GTE coatings.

The absorption spectrum of synthesized powders reveals characteristics of ZnO response, with a maximum around 360 nm (Figure 3). A strong and rather sharp absorption peak is recorded; thus, a photocatalytic effect on such a wavelength may be expected. Deposited films show a much lower absorption response with not so pronounced maxima, which may be a repercussion of the measurement method, since the specific surface of powders in the dispersion is much larger than in the films, considering the transmission of the laser path through the cuvette, unlike the simple reflection when the measuring of the films is performed. In addition, a strong influence of the alumina substrate [49] is observed due to the UV–vis device spot size also covering the bare substrate. It is probable that the strongest absorption may be from the substrate itself, below 250 nm (Figure 4).

Further analysis revealed that powders show expected values of bandgaps of 3.18 eV and 3.16 eV for ZnO/BPE and ZnO/GTE, respectively. Such values are in general agreement for ZnO nanoparticles [50,51], and are later proven as being effective in photocatalysis. On the other hand, when deposited, the photocatalytic activity of films was rather low due to a bandgap shift to much higher values. More specifically, bandgaps for ZnO/BPE 30 and ZnO/BPE 60 films were 5.075 eV and 5.00 eV, whilst bandgaps for ZnO/GTE 30 and ZnO/GTE 60 films were 5.1 eV and 5.05 eV, respectively. The reason for such an increase in bandgap can be related to the fact that the deposition of ZnO paste by the doctor blade or screen-printing technique requires additional additives, such as ethyl cellulose, that may cover the surface of the film and give a dominant signal. Another possible reason was the abovementioned strong response of Al_2_O_3_ substrates and overlapping ZnO, but, nevertheless, the low absorption of deposited films is evident.

### 3.2. Photocatalytic Activity of ZnO/BPE Coatings

**Figure 5:**
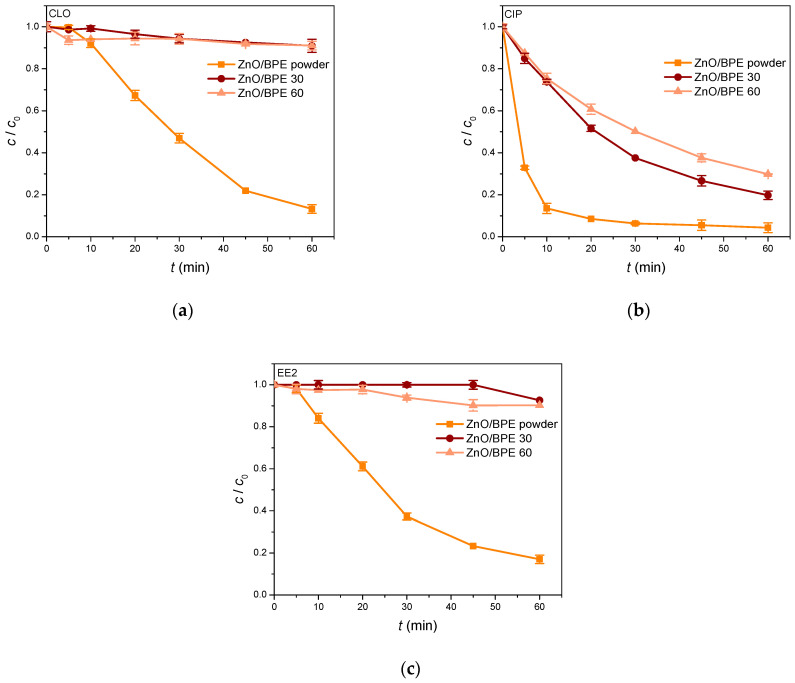
Photocatalytic degradation of (**a**) CLO; (**b**) CIP; and (**c**) EE2 (0.05 mmol/dm^3^) in the presence of ZnO/BPE 30 and ZnO/BPE 60 coatings under SSI.

The photocatalytic activity of ZnO/BPE 30 and ZnO/BPE 60 was compared to that of the green nanopowder previously synthesized by our research group [34]. In the case of all studied pollutants, a reduced removal efficiency was noticed in the presence of novel coatings, yet to varying extents (Figure 5). It is particularly emphasized in the case of CLO and EE2, when, after 60 min of SSI, less than 10% was removed in both respects. On the other hand, two coatings proved to have a satisfying photocatalytic activity when CIP was used as a model pollutant. Namely, high CIP removal efficiencies of 80.2 and 70.1% were reached with ZnO/BPE 30 and ZnO/BPE 60, respectively. This reduction in the removal efficiency may be caused by the low surface contact between the immobilized catalyst and pollutant molecules, compared to powders [52]. Additionally, lower efficiency in general may be caused by the presence of binders in films, necessary for film mechanical properties, without sintering/exposure to higher temperatures while processing.

### 3.3. Photocatalytic Activity of ZnO/GTE Coatings

**Figure 6:**
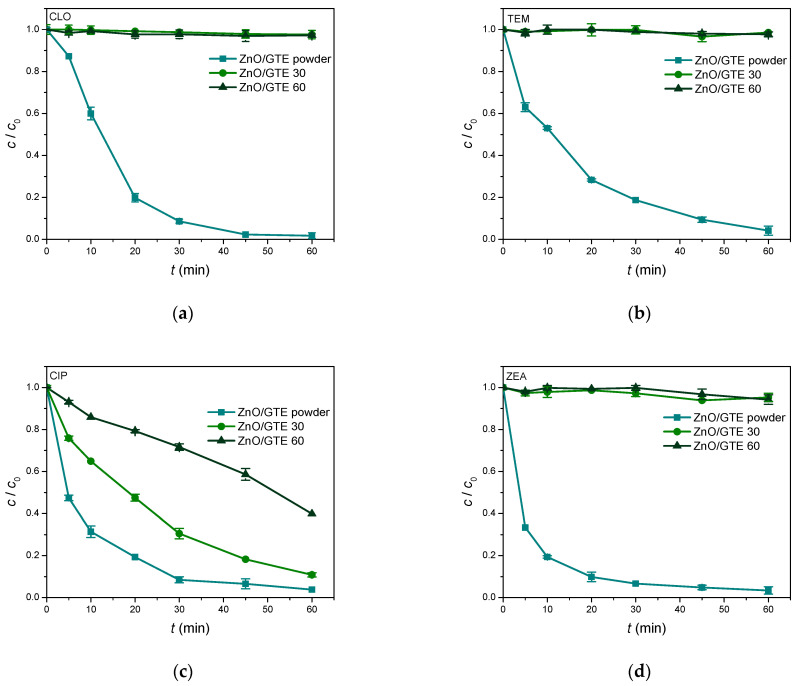
Comparative photocatalytic degradation of (**a**) CLO; (**b**) TEM; (**c**) CIP (0.05 mmol/dm^3^); and (**d**) ZEA (0.5 μg/cm^3^) in the presence of ZnO/GTE 30 and ZnO/GTE 60 coatings under SSI.

Figure 6 represents the comparison of the photocatalytic activity of two prepared ZnO/GTE coatings and selected nanomaterial, previously prepared by our research group [23], in the removal of two herbicides, one pharmaceutical, and one mycotoxin. Analogous to the previous case, overall lower removal efficiency of all four target molecules was observed in the presence of two new coatings. As previously mentioned, this is probably due to reduced surface contact between the catalyst and pollutant molecules [52], and assumptions related to the binder effect used for film deposition. However, once again, in the case of one pharmaceutical, CIP, a noticeable removal efficiency was achieved after 60 min of SSI: 89.1% in the presence of ZnO/GTE 30 and 60.2% using ZnO/GTE 60, respectively. It appears that the aforementioned rough surface of ZnO/GTE was beneficial for the photocatalytic process of CIP.

### 3.4. Reutilization of ZnO/GTE 30 Coating

**Figure 7:**
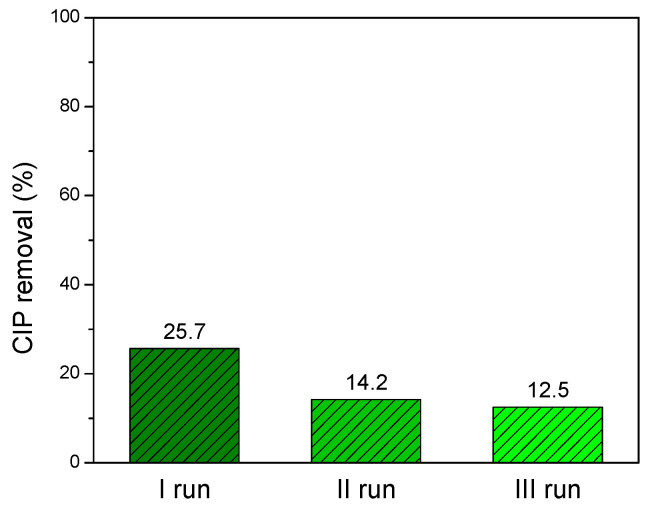
Reusability assessment of the ZnO/GTE 30 coating over three consecutive cycles for the solar-driven degradation of CIP (0.05 mmol/dm^3^) in river water.

A key aspect of heterogeneous photocatalysis is the ability to reuse the same photocatalyst multiple times while maintaining similar or slightly reduced pollutant removal efficiency. The results of the reutilization experiments for ZnO/GTE 30 coating, conducted under SSI across three consecutive photocatalytic degradation runs of CIP in Danube River water, are presented in Figure 7. The first observation to be made is the severe reduction in the final removal efficiency of CIP caused by the replacement of ultrapure with river water (89.1% → 25.7%). It can be due to various cations (predominantly calcium and magnesium) and anions (mainly chlorides and sulfates), as well as organic matter (e.g., humic acid) present in the river water [34,53]. Namely, the aforementioned species can adsorb onto the surface and block active sites, causing spatial deactivation through the reduction in the interaction between CIP molecules and the coating surface [54].

Observation regarding the durability and reusability of ZnO/GTE 30 coating indicates that similar removal efficiencies were obtained after three consecutive photocatalytic runs. Still, the slight decrease in the removal efficiency could be caused by the saturation of the photocatalyst surface, in the case of the cleaning process not being enough to remove the residues of CIP and intermediaries’ molecules. A comparable trend was observed in other studies [55,56,57].

## 4. Conclusions and Outlooks

This research sought to prepare eco-friendly coatings for the degradation of emerging organic pollutants. Structural characterization by XRD has confirmed that green ZnO-based powders successfully kept their hexagonal wurtzite crystal structure in all four prepared coatings. HRSEM of the films indicated rod-like particles up to 120 nm for ZnO/BPE coatings, whereas two ZnO/GTE coatings consisted of smaller, spherical shaped particles with diameters 30–70 nm. Bandgap analysis by UV–vis spectrometry indicated that powders did have values expected for ZnO nanomaterials, but films had shown much higher values for bandgaps (~5 eV), which was reflected in pollutant removal efficiency. The reason behind that was probably a lower film-specific surface, and the presence of binder in the films. After that, the photocatalytic activity of the newly prepared ZnO-based coatings was put to test in the photocatalytic removal of two pesticides (CLO and TEM), two pharmaceuticals (CIP and EE2), and one mycotoxin (ZEA). Satisfying removal efficiency was observed only in the case of CIP (80.2% with ZnO/BPE 30 and 89.1% with ZnO/GTE 30). Having outperformed other coatings, the reutilization of ZnO/GTE 30 was investigated within three consecutive runs removing CIP from the Danube River water after 60 min of SSI. Even though CIP removal efficiency was under 30%, presumably caused by the competition of its molecules with various species for the active sites on the photocatalyst surface, ZnO/GTE 30 coating exhibited satisfactory reusability and robustness. On the other hand, since coatings provided insufficient removal efficiency of CLO, TEM, EE2, and ZEA, certain improvements have to be made in the future in order to improve the removal of more resistant pollutants.

## Figures and Tables

**Table 1:** Key information about investigated organic pollutants [35,36,37,38,39].

Pollutant	Molecular Weight (g/mol)	Chemical Formula	Chemical Structure
CLO	239.70	C_12_H_14_ClNO_2_	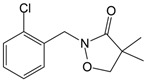
EE2	296.40	C_20_H_24_O_2_	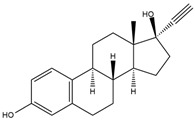
ZEA	318.37	C_18_H_22_O_5_	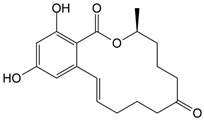
CIP	331.34	C_17_H_18_FN_3_O_3_	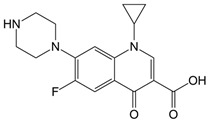
TEM	440.80	C_17_H_16_ClF_3_O_6_S	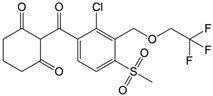

## Data Availability

The original contributions presented in this study are included in the article. Further inquiries can be directed to the corresponding authors.

## References

[1] Statista: Global Environmental Pollution—Statistics & Facts. https://www.statista.com/topics/4739/environmental-pollution/.

[2] Liu Z., Roosh M., Lu M., Arshad A., Xian W., Shen Y., Liu G., Bahadur A., Iqbal S., Mahmood S. (2025). Empowering wastewater treatment with step scheme heterojunction ternary nanocomposites for photocatalytic degradation of nitrophenol. Sci. Rep..

[3] Khan R., Anwar F., Ghazali F.M. (2024). A comprehensive review of mycotoxins: Toxicology, detection, and effective mitigation approaches. Heliyon.

[4] Van Scoy A.R., Tjeerdema R.S. (2014). Environmental fate and toxicology of clomazone. Rev. Environ. Contam. Toxicol..

[5] Du P., Wu X., Xu J., Dong F., Liu X., Zhang Y., Zheng Y. (2018). Clomazone influence soil microbial community and soil nitrogen cycling. Sci. Total Environ..

[6] Bognár S., Jovanović D., Putnik P., Despotović V., Ivetić T., Bajac B., Tóth E., Finčur N., Maksimović I., Putnik-Delić M. (2024). Solar-driven removal of selected organics with binary ZnO based nanomaterials from aquatic environment: Chemometric and toxicological assessments on wheat. J. Environ. Chem. Eng..

[7] Barchanska H., Kluza A., Krajczewska K., Maj J. (2015). Degradation study of mesotrione and other triketone herbicides on soils and sediments. J. Soils Sediments.

[8] Lazarević M., Putnik P., Šojić Merkulov D. (2022). Chemometric evaluation of different parameters for removal of tembotrione (agricultural herbicide) from water by adsorption and photocatalytic degradation using sustainable nanotechnology. Food Energy Secur..

[9] Wang X., Wen S., Shi T., Li Q.X., Lv P., Hua R. (2022). Photocatalysis of the triketone herbicide tembotrione in water with bismuth oxychloride nanoplates: Reactive species, kinetics and pathways. J. Environ. Chem. Eng..

[10] Brunner M., Langer O., Dobrozemsky G., Muller U., Zeitlinger M., Mitterhauser M., Wadsak W., Dudczak R., Kletter K., Muller M. (2004). [18F]Ciprofloxacin, a new positron emission tomography tracer for noninvasive assessment of the tissue distribution and pharmacokinetics of ciprofloxacin in humans. Antimicrob. Agents Chemother..

[11] Hopanna M., Mangalgiri K., Ibitoye T., Ocasio D., Snowberger S., Blaney L., Hernández-Maldonado A.J., Blaney L. (2019). UV-254 transformation of antibiotics in water and wastewater treatment processes. Contaminants of Emerging Concern in Water and Wastewater: Advanced Treatment Processes.

[12] Naslund J., Hedman J.E., Agestrand C. (2008). Effects of the antibiotic ciprofloxacin on the bacterial community structure and degradation of pyrene in marine sediment. Aquat. Toxicol..

[13] Cappelli F., Longoni O., Rigato J., Rusconi M., Sala A., Fochi I., Palumbo M.T., Polesello S., Roscioli C., Salerno F. (2022). Suspect screening of wastewaters to trace anti-COVID-19 drugs: Potential adverse effects on aquatic environment. Sci. Total Environ..

[14] Dominguez-Garcia P., Rodriguez R.R., Barata C., Gomez-Canela C. (2023). Presence and toxicity of drugs used to treat SARS-CoV-2 in Llobregat River, Catalonia, Spain. Environ. Sci. Pollut. Res. Int..

[15] Peng L., Dai X., Liu Y., Sun J., Song S., Ni B.J. (2018). Model-based assessment of estrogen removal by nitrifying activated sludge. Chemosphere.

[16] Aoki J.Y., Hatsuyama A., Hiramatsu N., Soyano K. (2011). Effects of ethynylestradiol on vitellogenin synthesis and sex differentiation in juvenile grey mullet (*Mugil cephalus*) persist after long-term exposure to a clean environment. Comp. Biochem. Physiol. Part-C Toxicol. Pharmacol..

[17] Reyhanian N., Volkova K., Hallgren S., Bollner T., Olsson P.E., Olsen H., Hallstrom I.P. (2011). 17alpha-ethinyl estradiol affects anxiety and shoaling behavior in adult male zebra fish (*Danio rerio*). Aquat. Toxicol..

[18] Aris A.Z., Shamsuddin A.S., Praveena S.M. (2014). Occurrence of 17alpha-ethynylestradiol (EE2) in the environment and effect on exposed biota: A review. Environ. Int..

[19] Schirling M., Bohlen A., Triebskorn R., Kohler H.R. (2006). An invertebrate embryo test with the apple snail Marisa cornuarietis to assess effects of potential developmental and endocrine disruptors. Chemosphere.

[20] Zhang G.L., Feng Y.L., Song J.L., Zhou X.S. (2018). Zearalenone: A mycotoxin with different toxic effect in domestic and laboratory animals’ granulosa cells. Front. Genet..

[21] Švarc-Gajić J., Brezo-Borjan T., Jakšić S., Despotović V., Finčur N., Bognár S., Jovanović D., Šojić Merkulov D. (2024). Decomposition of organic pollutants in subcritical water under moderate conditions. Processes.

[22] Dhamorikar R.S., Lade V.G., Kewalramani P.V., Bindwal A.B. (2024). Review on integrated advanced oxidation processes for water and wastewater treatment. J. Ind. Eng. Chem..

[23] Bognár S., Jovanović D., Despotović V., Jakšić S., Panić S., Milanović M., Finčur N., Putnik P., Šojić Merkulov D. (2025). Advanced photocatalytic degradation of organic pollutants using green tea-based ZnO nanomaterials under simulated solar iradiation in agri-food wastewater. Foods.

[24] Fujishima A., Honda K. (1972). Electrochemical photolysis of water at a semiconductor electrode. Nature.

[25] Lee K.M., Lai C.W., Ngai K.S., Juan J.C. (2016). Recent developments of zinc oxide based photocatalyst in water treatment technology: A review. Water Res..

[26] Parihar V., Raja M., Paulose R. (2018). A brief review of structural, electrical and electrochemical properties of zinc oxide nanoparticles. Rev. Adv. Mater. Sci..

[27] Comparelli R., Fanizza E., Curri M.L., Cozzoli P.D., Mascolo G., Agostiano A. (2005). UV-induced photocatalytic degradation of azo dyes by organic-capped ZnO nanocrystals immobilized onto substrates. Appl. Catal. B Environ..

[28] Bognár S., Putnik P., Šojić Merkulov D. (2022). Sustainable green nanotechnologies for innovative purifications of water: Synthesis of the nanoparticles from renewable sources. Nanomaterials.

[29] Sirelkhatim A., Mahmud S., Seeni A., Kaus N.H.M., Ann L.C., Bakhori S.K.M., Hasan H., Mohamad D. (2015). Review on zinc oxide nanoparticles: Antibacterial activity and toxicity mechanism. Nano-Micro Lett..

[30] Verma R., Pathak S., Srivastava A.K., Prawer S., Tomljenovic-Hanic S. (2021). ZnO nanomaterials: Green synthesis, toxicity evaluation and new insights in biomedical applications. J. Alloys Compd..

[31] Abdel-Maksoud Y., Imam E., Ramadan A. (2016). TiO_2_ solar photocatalytic reactor systems: Selection of reactor design for scale-up and commercialization—Analytical review. Catalysts.

[32] Anwer H., Mahmood A., Lee J., Kim K.-H., Park J.-W., Yip A.C.K. (2019). Photocatalysts for degradation of dyes in industrial effluents: Opportunities and challenges. Nano Res..

[33] Navidpour A.H., Xu B., Ahmed M.B., Zhou J.L. (2024). Immobilization of TiO_2_ and ZnO by facile surface engineering methods to improve semiconductor performance in photocatalytic wastewater treatment: A review. Mater. Sci. Semicond..

[34] Jovanović D., Bognár S., Despotović V., Finčur N., Jakšić S., Putnik P., Deák C., Kozma G., Kordić B., Šojić Merkulov D. (2024). Banana peel extract-derived ZnO nanopowder: Transforming solar water purification for safer agri-food production. Foods.

[35] PubChem: Clomazone. https://pubchem.ncbi.nlm.nih.gov/compound/54778.

[36] PubChem: 17α-Ethinylestradiol. https://pubchem.ncbi.nlm.nih.gov/compound/5991.

[37] PubChem: Zearalenone. https://pubchem.ncbi.nlm.nih.gov/compound/5281576.

[38] PubChem: Ciprofloxacin. https://pubchem.ncbi.nlm.nih.gov/compound/2764.

[39] PubChem: Tembotrione. https://pubchem.ncbi.nlm.nih.gov/compound/Tembotrione.

[40] Xu C., Willenbacher N. (2018). How rheological properties affect fine-line screen printing of pastes: A combined rheological and high-speed video imaging study. J. Coat. Technol. Res..

[41] Tauc J. (1968). Optical properties and electronic structure of amorphous Ge and Si. Mater. Res. Bull..

[42] Kubelka P., Munk F. (1931). An article on optics of paint layers. Z. Techn. Physik..

[43] Köferstein R., Jäger L., Ebbinghaus S.G. (2013). Magnetic and optical investigations on LaFeO_3_ powders with different particle sizes and corresponding ceramics. Solid State Ion..

[44] Samardžić N.M., Bajac B., Bajić J., Đurđić E., Miljević B., Srdić V.V., Stojanović G.M. (2018). Photoresistive switching of multiferroic thin film memristors. Microelectron. Eng..

[45] Miljević B., van der Bergh J.M., Vučetić S., Lazar D., Ranogajec J. (2017). Molybdenum doped TiO_2_ nanocomposite coatings: Visible light driven photocatalytic self-cleaning of mineral substrates. Ceram. Int..

[46] Jovanović D., Orčić D., Šojić Merkulov D., Despotović V., Finčur N. (2025). Study of factors impacting the light-driven removal of ciprofloxacin (Ciprocinal^®^) and identification of degradation products using LC–ESI–MS^2^. J. Photochem. Photobiol..

[47] Šojić Merkulov D., Vlazan P., Poienar M., Bognár S., Ianasi C., Sfirloaga P. (2023). Sustainable removal of 17α-ethynylestradiol from aqueous environment using rare earth doped lanthanum manganite nanomaterials. Catal. Today.

[48] Hassan A., Jalil A., Ilyas S.Z., Iqbal M.F., Ali Shah S.Z., Baqir Y. (2024). Green-route synthesis and ab-initio studies of a highly efficient nano photocatalyst:Ce/zinc-oxide nanopetals. Heliyon.

[49] Bharthasaradhi R., Nehru L.C. (2015). Structural and phase transition of α-Al_2_O_3_powders obtained by co-precipitation method. Ph. Transit..

[50] Abdel-Salam A.I., Soliman T.S., Khalid A., Awad M.M., Abdallah S. (2024). Effect of reduced graphene oxide on the structural and optical properties of ZnO nanoparticles. Mater. Lett..

[51] Afzal H., Ikram M., Ali S., Shahzadi A., Aqeel M., Haider A., Imran M., Ali S. (2020). Enhanced drug efficiency of doped ZnO–GO (graphene oxide) nanocomposites, a new gateway in drug delivery systems (DDSs). Mater. Res. Express.

[52] Kassegn Weldegebrieal G., Kassegn Sibhatu A., Shah M. (2022). Photocatalytic degradation of organic contaminants in wastewater treatment. Environmental Microbiology.

[53] Jevtić I., Jakšić S., Šojić Merkulov D., Bognár S., Abramović B., Ivetić T. (2023). Matrix effects of different water types on the efficiency of fumonisin B1 removal by photolysis and photocatalysis using ternary- and binary-structured ZnO-based nanocrystallites. Catalysts.

[54] Rajbongshi B.M., Hussain C.M., Kumar Mishra A. (2020). Photocatalyst: Mechanism, challenges, and strategy for organic contaminant degradation. Handbook of Smart Photocatalytic Materials.

[55] Finčur N.L., Grujić-Brojčin M., Šćepanović M.J., Četojević-Simin D.D., Maletić S.P., Stojadinović S., Abramović B.F. (2021). UV-driven removal of tricyclic antidepressive drug amitriptyline using TiO_2_ and TiO_2_/WO_3_ coatings. React. Kinet. Mech. Catal..

[56] Topkaya E., Konyar M., Yatmaz H.C., Ozturk K. (2014). Pure ZnO and composite ZnO/TiO_2_ catalyst plates: A comparative study for the degradation of azo dye, pesticide and antibiotic in aqueous solutions. J. Colloid Interface Sci..

[57] Lim N.Y.Y., Chiam S.L., Leo C.P., Pung S.Y., Chang C.K., Ang W.L. (2025). Suppression of charge recombination using microfibrillated cellulose in carboxymethyl cellulose coatings containing ZnO nanorods and BiOCl during photoelectrocatalysis. Int. J. Biol. Macromol..

